# Decisional Procrastination in Academic Settings: The Role of Metacognitions and Learning Strategies

**DOI:** 10.3389/fpsyg.2017.00973

**Published:** 2017-06-16

**Authors:** Valeria de Palo, Lucia Monacis, Silvana Miceli, Maria Sinatra, Santo Di Nuovo

**Affiliations:** ^1^Department of Human Sciences, University of FoggiaFoggia, Italy; ^2^Department of Psychological, Pedagogical, and Educational Sciences, University of PalermoPalermo, Italy; ^3^Department of Educational Sciences, Psychology, Communication, University of Bari Aldo MoroBari, Italy; ^4^Department of Educational Sciences, University of CataniaCatania, Italy

**Keywords:** decisional procrastination, metacognitive beliefs, learning strategies, anxiety, time management

## Abstract

Nowadays, university students suffer from a broad range of problems, such as educational underachievement or the inability to control themselves, that lead to procrastination as a consequence. The present research aimed at analyzing the determinants of decisional procrastination among undergraduate students and at assessing a path model in which self regulated learning strategies mediated the relationship between metacognitive beliefs about procrastination and decisional procrastination. 273 students from Southern Italy filled out a questionnaire composed by: the socio-demographic section, the Metacognitive Beliefs About Procrastination Questionnaire, the procrastination subscale of the Melbourne Decision Making Questionnaire, and the Anxiety, the Time Management, and the Information Processing subscales of the Learning and Study Strategies Inventory. Results showed that the relationship between negative and positive metacognitive beliefs about procrastination and decisional procrastination was mediated only by time management and anxiety. Such findings underlined the crucial role played by learning strategies in predicting the tendency to delay decisional situations and in mediating the relationship between metacognitive beliefs about procrastination and decisional procrastination.

## Introduction

Nowadays, university students suffer from a broad range of problems, such as educational underachievement, the inability to control themselves, subjective discomfort. The consequence may be the tendency to postpone academic tasks and/or decisions on academic career. This tendency has been generally conceptualized in terms of procrastination, a construct that has been differently understood.

First, procrastination has been used for either dysfunctional forms (e.g., Steel, 2007) or positive or strategic forms of delay (Chu and Choi, 2005), although it is *per se* dysfunctional because it implies an unnecessary delay and negative consequences with regard to performance and subjective well-being (Klingsieck, 2013a,b). Second, the construct has been connoted according to the assumed theoretical perspectives. From the differential psychology perspective, it has been considered a trait or behavioral disposition consisting in the tendency to delay the start or completion of a task because of the lack of self regulation (Milgram and Tenne, 2000). From the motivational and volitional perspective, procrastination has been regarded as a failure in motivation and volition, leading to the intention-action gap (Lay, 1986; Steel, 2007; Steel and Klingsieck, 2016). In this vein, it has been associated with self regulation (Dietz et al., 2007), self control (Schouwenburg and Groenewoud, 2001), action-control (Blunt and Pychyl, 2005), time management (Strunk et al., 2013), time orientation (Ferrari and Díaz-Morales, 2007), and learning strategies (Howell and Watson, 2007). With regard to the clinical perspective, the focus is on the clinically relevant extent of procrastination and on the link between procrastination and depression (Flett et al., 1995), or anxiety (Spada et al., 2006). Finally, the situational perspective has dealt with the situational features pertaining to task characteristics, such as task difficulty and attractiveness (Blunt and Pychyl, 2000), plausibility of the assignment (Milgram et al., 1992). Additional approaches have tried to link procrastination with parenting styles (Pychyl et al., 2002), biological explanations (Rabin et al., 2011) or extraversion according to the biologically-based theory (Freeman et al., 2011). In light of these manifold theoretical approaches, procrastination cannot be explained by one perspective alone: the integration of the different perspectives is required to achieve its concept and dynamics (Klingsieck, 2013b, p. 28).

A further distinction has concerned behavioral procrastination, decisional procrastination (Ferrari, 1994, 1998; Milgram and Tenne, 2000; Tibbett and Ferrari, 2015), arousal procrastination (i.e., procrastination due to the belief that one works best under pressure), and avoidance procrastination (i.e., procrastination due to imagined and actual fears) (Ferrari, 1992; Ferrari et al., 2009). Even though specific measures of these last types of procrastination have been questioned (Simpson and Pychyl, 2009; Steel, 2010), decisional procrastination is still an object of interest (e.g., Mirzaei et al., 2014; Fernie et al., 2016b).

Given the high prevalence of procrastination among university students with obvious consequences on academic success and satisfaction (e.g., Balkis et al., 2013; Balkis and Duru, 2015; Grunschel and Schopenhauer, 2015; Grunschel et al., 2016), the current study focused on the particular aspect of decisional procrastination, specifically intended by Janis and Mann (1977) as indecisiveness due to its reference to handling conflicts in decision-making situations. According to these authors’ “conflict-theory model of decision-making,” there are five patterns of coping with the stress caused by a difficult decision: *unconflicted adherence*, when the individual, ignoring information about the risks, decides to continue his/her action; *unconflicted change*, when the individual uncritically adopts the most salient or recommended action; *vigilance*, when the individual, facing an option that has risks and having sufficient time, hopes to find the better solution among alternatives; *hypervigilance*, when the individual, triggered by the time pressure, makes a decision in response to approaching deadlines without considering all of the available alternatives; *defensive avoidance*, when the individual escapes conflict by procrastinating, thus making faulty decisions. The last pattern foresees, in turn, three types of coping: procrastinating, shifting the responsibility of the decision to someone else, and selecting the least objectionable course of action.

The first defensive avoidance pattern, namely decisional procrastination, represents one of the most problematic issues the students have to deal with when they have to make decisions on their academic tasks (Mann, 2016). The existing few studies reported negative correlations between buckpassing, procrastination, hypervigilance decision-making styles, and students’ life satisfaction (Deniz, 2006; Balkis, 2013), and negative effects of these non-adaptive patterns of coping on students’ well-being (Yilmaz et al., 2013).

Moreover, since procrastination has been generally assumed as a form of self regulation failure (Baumeister et al., 1994; Harriott and Ferrari, 1996; Stöber and Joormann, 2001), in this study self regulation in learning was hypothesized to be an antecedent of decisional procrastination. Such an hypothesis was based on Zimmerman and Schunk’s (1989) conceptualization that self regulated learning (SRL) refers to students’ self generated thoughts, feelings, and actions oriented toward the achievement of their goals.

Due to the main role of self regulated learning strategies in predicting students’ academic weaknesses or strengths (Zimmerman and Schunk, 2001; Cano, 2006; Tan et al., 2008), Weinstein et al. (1987) theoretical approach to strategic learning was assumed in the current research. The model foresaw three basic components: *Skill*, that includes Information Processing, Selecting Main Ideas, and Test Strategies; *Will*, that includes Anxiety, Attitude, and Motivation; and *Self regulation*, that includes Concentration, Self testing, Study Aids, and Time Management (Weinstein and Palmer, 2002, pp 4–6).

To the aim of the present study, one indicator of each component was chosen because of its consistent theoretical relationship with the *meaning* of procrastination, that is:

– Information processing: how students are able to use imagery, verbal elaboration, organization strategies, and reasoning skills as learning strategies in order to learn new information and skills and to build bridges between what they already know and what they are trying to learn and remember;
– Anxiety: the degree to which students worry about their academic performance;
– Time management: how students use time management principles for academic tasks.

Metacognitive belief about procrastination (Fernie and Spada, 2008) was also investigated. The construct was extrapolated from Wells and Matthews’ metacognitive theory of emotional disorders. Accordingly, metacognitive beliefs were intended as the information individuals hold about their own cognition and internal states, and about the coping strategies that impact on both (Wells and Matthews, 1994, 1996; Wells, 2000). Metacognitive beliefs were implicated in various psychological problems, from anxiety (Spada et al., 2008) to depression (Papageorgiou and Wells, 2003), behavioral and decisional procrastination (Spada et al., 2006). In light of the findings emerged from Spada et al. (2006) research, Fernie and Spada (2008) identified: (1) positive metacognitive beliefs about procrastination, referred to the individual’s belief that procrastination is useful in improving cognitive performance, and (2) negative metacognitive beliefs about procrastination, referred to the uncontrollability of procrastination. Both positive and negative metacognitive beliefs were described as maladaptive coping strategies. A link between decisional procrastination, negative affect, metacognitive beliefs, and attentional control factors has been recently demonstrated (Fernie et al., 2016b) using the Metacognitive Beliefs About Procrastination Questionnaire (Fernie et al., 2009).

To better understand the determinants of decisional procrastination in a sample of undergraduate students, the current research intended to assess a path model in which self regulated learning strategies mediated the relationship between metacognitive beliefs about procrastination and decisional procrastination. Specifically, it was expected that: (1) positive and negative metacognitive beliefs determined higher levels of anxiety and decisional procrastination, and lower levels of time management and informational processing; (2) higher scores of time management and information processing determined lower scores of decisional procrastination, whereas anxiety determined higher levels of decisional procrastination; (3) time management, information processing, and anxiety mediated the relationship between metacognitive beliefs and decisional procrastination.

## Materials and Methods

### Participants

The initial sample was composed by 297 undergraduate students recruited from universities through convenience sampling. The inclusion criterion was being a fluent speaker of Italian. 24 participants were excluded because they did not complete the questionnaire. The final sample consisted of 273 Italian participants (*M*_age_ = 22.16, *SD* = 3.97; 254 were females).

### Procedures

Data collection took place from April to June 2016. The respondents were voluntary invited to participate in the research by completing individually a battery of anonymous self report questionnaires in approximately 20 min during an ordinary 50-min classroom lesson. Potential order effects were controlled by presenting the scales of the battery in three randomized orders. Respondents provided written informed consent. The study was conducted in accordance with the ethical principles of the Declaration of Helsinki for conducting research with human participants. The protocol was reviewed and approved by the local institutional independent ethics committee.

### Measures

The English versions of the instruments were translated into Italian separately by the Italian authors of the present study. After the measures were translated into Italian, they were back-translated into English by a native speaker to establish their comparability.

The degree to which students worry about academic performance, apply time management principles to academic situations, and use organizational strategies and reasoning skills were assessed through the Italian translation of three subscales of the *Learning and Study Strategies Inventory* (LASSI – 2nd Edn, Weinstein and Palmer, 2002; 1st Edn, Weinstein et al., 1987): *Anxiety* (ANX), *Time Management* (TM), and *Information Processing* (IP). Sample items are “When I am taking a test, worrying about doing poorly interferes with my concentration” (ANX), “I put off studying more than I should” (TM), “I try to find relationships between what I am learning and what I already know” (IP). Each subscale is comprised of 10 items rated on a five-point Likert scale (from 1 = *Not at all typical of me* to 5 = *Very much typical of me*). Low scores in the ANX, TM, and IP subscales indicate high levels of anxiety, difficulty in the use of time management techniques and in the organization of what the students are trying to learn. The internal consistency was found to be satisfactory for the three subscales (Cronbach’s alpha = 0.83 for ANX, 0.70 for IP, and 0.68 for TM, respectively).

The *Metacognitive Beliefs about Procrastination Questionnaire* (MCPQ; Fernie et al., 2009) is composed by two dimensions assessing positive and negative metacognitive beliefs about procrastination. Each dimension consists of eight items rated on a four-point Likert scale (from 1 = *Do not agree* to 4 = *Agree very much*). Sample items are “Procrastination allows creativity to occur more naturally” (Positive beliefs), “Procrastination makes me feel down” (Negative beliefs). Higher scores on both dimensions indicate higher levels of maladaptation in metacognitive beliefs. The internal consistencies were satisfactory (Cronbach’s alpha = 0.73 for the Positive beliefs dimension and 0.84 for the Negative beliefs dimension).

Decisional procrastination was assessed by using the *Procrastination* subscale of the *Melbourne Decision-Making Questionnaire* – Italian version (MDMQ; Mann et al., 1997; Nota and Soresi, 2000). The subscale includes five items rated on a five-point Likert scale (from 1 = N*ot true for me* to 5 = *True for me*). Sample item is “I waste a lot of time on trivial matters before getting to the final decision.” The total score was computed by averaging the items. Higher scores indicate the tendency to postpone the moment in which individuals have to cope with decisional problems. The scale reliability was good (Cronbach’s alpha = 0.81).

### Analysis Strategies

Descriptive statistics included minimum, maximum, mean, and standard deviation of the scores of each scale. Preliminary data analyses included screening for missing data and outliers, as well as assessing for normality. No missing data were found. The univariate normality of the scores was checked through skewness and kurtosis values. The univariate outliers were identified using the graphic approach (inspection of Boxplot), whereas the Mahalanobis Distance analysis and the critical value based on the chi-square distribution values were used to identify multivariate outliers. The pattern of associations between the variables of interest were assessed using bivariate correlations.

Path analysis with observed variables was carried out to test the theoretically-predicted model, which assumed decisional procrastination as dependent variable, positive and negative metacognitive beliefs about procrastination as correlated independent variables, and information processing, anxiety and time management as mediator variables. The following goodness-of-fit indices were used: the chi-squared (χ^2^) statistic and its degree of freedom; the Root Mean Square Error of Approximation (RMSEA) and its 90% confidence interval (90% CI); the Comparative Fit Index (CFI); and the Standardized Root Mean Square Residuals (SRMR). For CFI, values greater than or equal to 0.90 indicated a good fit; values greater than or equal to 0.95 indicated an excellent fit. RMSEA and SRMR values of 0.08 or less indicated an adequate fit, whereas values of 0.06 or less reflected a good fit (Browne and Cudeck, 1993; Hu and Bentler, 1999). Direct and indirect relationships were tested. Analyses were performed using MPlus 7.0 and SPSS 20.0 for Windows.

## Results

Descriptive statistics, including minimum, maximum, mean, and standard deviation of each variable taken into account are reported in **Table 1**.

**Table 1:** Descriptive statistics of the variables of interest.

	Minimum–maximum	Mean (*SD*)	Skewness	Kurtosis
Anxiety	8–40	21.02 (6.69)	0.358	-0.132
Information processing	18–40	29.91 (4.51)	0.051	-0.284
Time management	16–40	28.71 (4.81)	-0.255	-0.050
Negative beliefs about procrastination	9–32	21.07 (5.07)	-0.212	-0.350
Positive beliefs about procrastination	8–32	19.41 (4.04)	-0.166	0.278
Decisional procrastination	1–3	1.24 (0.46)	0.824	0.457

Bivariate correlations were performed to explore the associations between the scores of the anxiety, information processing, time management subscales, the metacognitive beliefs about procrastination dimensions, and the decisional procrastination scale. Results are showed in **Table 2**.

**Table 2:** Bivariate correlations between the variables of interest.

	Metacognitive beliefs about procrastination		Learning strategies		
	Positive beliefs	Negative beliefs	Anxiety	Information processing	Time management
Anxiety	-0.193^∗∗^	-0.165^∗∗^			
Information processing	0.023	0.223^∗∗∗^	-0.103		
Time management	-0.198^∗∗∗^	-0.026	0.163^∗∗^	0.205^∗∗∗^	
Decisional procrastination	0.229^∗∗∗^	0.066	-0.292^∗∗∗^	-0.155^∗^	-0.441^∗∗∗^

*^∗^p < 0.05; ^∗∗^ p < 0.01; ^∗∗∗^ p < 0.001*.

**FIGURE 1:**
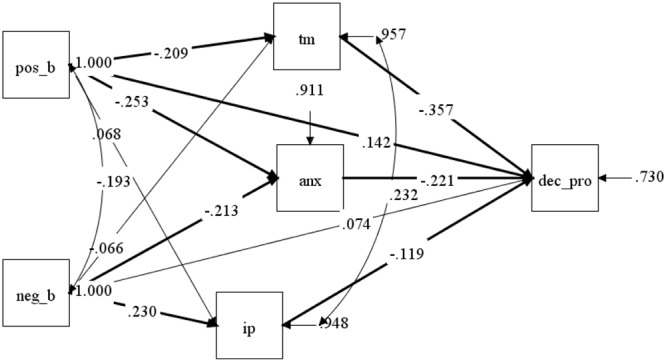
Path diagram of the relationship between metacognitive beliefs about procrastination, time management, anxiety, information processing, and decisional procrastination with standardized parameter estimates. **pos_b**, positive beliefs about procrastination; **neg_b**, negative beliefs about procrastination; **tm**, time management; **anx**, anxiety; **ip**, information processing; **dec_pro**, decisional procrastination. Significant paths (*p* < 0.05) are in bold.

Path analysis was used to test the hypothesized multivariate relations among the variables. The model assumed direct and indirect effects of metacognitive beliefs about procrastination on decisional procrastination via self regulated learning strategies (anxiety, information processing, and time management). The fit indices of the first model were not adequate, χ^2^ = 21.429, df = 3, *p* < 0.001; RMSEA = 0.148, 90% C.I. = 0.093–0.210; CFI = 0.88; SRMR = 0.052. The model was re-specified on the basis of the modification indices by removing step-by-step the non-significant paths, i.e., between negative beliefs, TM, and decisional procrastination, and between positive beliefs and IP. Moreover, a careful inspection of the modification indices indicated that the model fit indices could improve if TM and IP were allowed to correlate. Such a modification was justified by the positive correlation between the two dimensions reported by Weinstein and Palmer (2002, p. 27). The second model showed better fit indices, χ^2^ = 10.345, df = 5, *p* = 0.06; RMSEA = 0.062, 90% C.I. = 0.000–0.115; CFI = 0.97; TLI = 0.90; SRMR = 0.039. As expected, decisional procrastination was predicted negatively by TM and IP, and positively by ANX and positive beliefs about procrastination; positive beliefs predicted negatively ANX and TM, whereas negative beliefs about procrastination predicted negatively ANX and positively IP. As for the indirect effects, decisional procrastination was predicted by positive beliefs via TM and ANX, and by negative beliefs only via ANX (**Table 3**). The model explained 26.8% of the variance of decisional procrastination, 8.9% of the variance of anxiety, 5.4% of the variance of information processing, and 4.5% of the variance of time management. The final path diagram is shown in **Figure 1**.

**Table 3:** Indirect effects.

	Estimate	*p*-value
**Effects from POS_B to DEC_PRO**		
Total	0.259	0.000
Total indirect	0.136	0.000
**Specific indirect**		
POS_B on DEC_PRO via TM	0.077	0.001
POS_B on DEC_PRO via ANX	0.059	0.002
Direct POS_B on DEC_PRO	0.123	0.020
**Effects from NEG_B to DEC_PRO**		
Total	0.026	0.223
Total indirect	0.026	0.223
**Specific indirect**		
NEG_B on DEC_PRO via ANX	0.050	0.005
NEG_B on DEC_PRO via IP	-0.024	0.079

*Standardized estimates. POS_B, positive beliefs about procrastination; NEG_B, negative beliefs about procrastination; ANX, anxiety; TM, time management; IP, information processing; DEC_PRO, decisional procrastination*.

## Discussion

The current study aimed at analyzing the relationships between metacognitive beliefs about procrastination, learning strategies, and decisional procrastination by means of path analysis. Findings confirmed the mediational model, even though the hypothesized relationships between positive beliefs and information processing, as well as between negative beliefs and both time management and decisional procrastination were not supported.

As for the first hypothesis concerning the links between metacognitive beliefs about procrastination and self regulated learning strategies, data revealed that positive and negative beliefs predicted higher levels of anxiety (the negative coefficient was justified by the scoring procedure of the Anxiety scale, in which low scores indicated high levels of anxiety). Namely, the maladaptive cognitive mechanism according to which procrastination is believed to be a useful coping strategy as well as an uncontrollable tendency to delay, may have contributed to a higher level of students’ worry about their own academic performance. This relationship was theoretically consistent with the current conceptions of anxiety as negative thoughts, beliefs, and emotions about one’s abilities and intelligence, or about the likelihood of success that divert students’ attention away from the task (Weinstein and Palmer, 2002).

The observed negative effect of positive beliefs about procrastination on time management should be interpreted in terms of a greater difficulty in using time management strategies for learning, such as the creation of realistic plans and schedules, and in dealing with distractions, probably because the attention was fixed on the stable belief in the usefulness of procrastination. Positive metacognitions predicted also decisional procrastination, that is, beliefs in procrastination as a useful strategy to improve cognitive performance may directly foster the tendency to postpone decisions. The findings were in line with the results of prior studies (Spada et al., 2006; Fernie et al., 2009; de Palo et al., 2016). Indeed, the link between positive metacognitions and decisional procrastination could be explained either taking into account the role played by such beliefs in facilitating the activation of “internal reality-testing” or “mental problem-solving” routines, which would interfere with decision-making processes, thus leading to decisional procrastination (Fernie et al., 2016b, p. 4), or considering the similarity between the constructs, both defined a form of coping (Mann et al., 1997; Fernie and Spada, 2008).

As for negative metacognitions, data showed a positive relationship between negative beliefs and information processing, in contrast to the stated hypothesis. The causal process underlying this association could be due to the fact that such beliefs stimulate a verbal activity that fixes attention on procrastination and consumes executive resources necessary for increasing flexible control over thinking and coping (Fernie et al., 2009). The information processing refers to how well-students can create imaginal and verbal elaborations, organization strategies, and reasoning skills to foster understanding and recall (Weinstein and Palmer, 2002, p. 10). Consequently, negative metacognitions may lead to an increasing of a similar cognitive mechanism, even though it is referred to learning processes.

The second hypothesis on the causal links between learning strategies and decisional procrastination was totally supported. Specifically, as for the first relationship between time management and decisional procrastination, the delay of a decision within some specific frame was a result of students’ difficulty in planning and controlling the time needed to complete academic tasks efficiently. Procrastination seems to be therefore an underregulation form of self regulation, reflecting deficiencies in evaluating, structuring, and managing time (Rabin et al., 2011). As for the second relationship between anxiety and decisional procrastination, the results showed how the degree to which students worried about their academic performance determined the tendency to escape difficult decisions by procrastinating, as already highlighted by Haycock et al. (1998). As a matter of fact, the self defeating behavior activated by anxiety in academic situations interrupts the various steps taken by students toward completion of their tasks. As for the third relationship between information processing and decisional procrastination, students who were lacking in strategies useful to add meaning and information to their prior knowledge, experiences, attitudes, and to organize what they were trying to learn, found difficult to acquire new knowledge and understanding and use synthesis, inferential, and analytic reasoning skills. Hence, they spent a large amount of time in studying and, consequently, procrastinated decisional situations.

Finally, as for the last hypothesis, only time management and anxiety mediated the relationship between metacognitions and decisional procrastination. Specifically, the two learning strategies partially mediated the relationship between positive beliefs and decisional procrastination. A careful inspection of beta coefficients revealed that the indirect effect of time management was higher than the indirect effect of anxiety, and that the direct path from positive beliefs to decisional procrastination was the highest. Furthermore, only anxiety totally mediated the relationship between negative beliefs and decisional procrastination. These causal processes accounted for the crucial role played by the two components of learning strategies (will and self regulation) in explaining the influence of the metacognitive beliefs on the tendency to delay decisional situations. The indirect effect of information processing was not significant probably because of the weakness of the direct path between information processing and decisional procrastination.

The findings provided further support to the assumption that individuals’ engagement in maladaptive cognitions, leaving less mental assets to task initiation or completion, reinforces negative self efficacy beliefs, thus postponing the making of a decision (Fernie et al., 2016a).

Suggestions for counseling practices in academic contexts could be inferred from the current research. For instance, to enable students to successfully achieve their academic goals, educators should be aware of the potential individual differences (Monacis et al., 2016; Steel and Klingsieck, 2016) and difficulties that could cause the academic career dropout. A possible intervention could be focused on the reinforcement of mental resources in order to promote the development of self regulated learning strategies. University counseling services should be provided taking into account research-based suggestions to address specific and targeted programs. Such programs could help students to improve their time management skills to dealing with the amount of work and difficult tasks (de Palo et al., 2012; Pychyl and Flett, 2012; Yerdelen et al., 2016).

From a theoretical point of view, this study provided for the first time empirical evidence for an *integrated* model that included general students’ cognitive mechanisms and self regulated strategies related to learning processes, thus explaining in depth the maladaptive pattern of postponing a decision when faced with conflicts and choices.

Notwithstanding, some limitations should be noted. First, self reports biases and context effects may have contributed to errors in self report instruments. Second, larger and more representative samples should be employed to replicate the model using latent variables. Third, the generalizability of the findings may be limited by the prevalence of females in the sample. Future studies should overcome these limitations.

## Author Contributions

VdP and LM: substantial contributions to the conception and design of the work, acquisition of data, analysis, and interpretation of data. SM, MS, and SDN: drafting the work and revising it critically for important intellectual content and for final approval of the version to be published.

## Conflict of Interest Statement

The authors declare that the research was conducted in the absence of any commercial or financial relationships that could be construed as a potential conflict of interest.
